# Functional Characterization of Neurofilament Light Splicing and Misbalance in Zebrafish

**DOI:** 10.3390/cells9051238

**Published:** 2020-05-16

**Authors:** Doris Lou Demy, Maria Letizia Campanari, Raphael Munoz-Ruiz, Heather D. Durham, Benoit J. Gentil, Edor Kabashi

**Affiliations:** 1 Institut Imagine, UMR-1163 INSERM et Université Paris Descartes, Hôpital Universitaire Necker-Enfants Malades, 24, boulevard du Montparnasse, 75015 Paris, France; doris-lou.demy@pasteur.fr (D.L.D.); maria-letizia.campanari@institutimagine.org (M.L.C.); munraphael@gmail.com (R.M.-R.); 2 Sorbonne Universités Paris VI, UMR INSERM U 1127, CNRS 1127 UPMC, Institut du Cerveau et de la Moelle épinière—ICM, 75015 Paris, France; 3 Department of Neurology and Neurosurgery and Montreal Neurological Institute, McGill University, Montreal, QC H3A 2B4, Canada; heather.durham@mcgill.ca (H.D.D.); benoit.gentil@mcgill.ca (B.J.G.); 4 Department of Kinesiology and Physical Education McGill University, Montreal, QC H3A 2B4, Canada

**Keywords:** amyotrophic lateral sclerosis (ALS), neurofilament light (NEFL), TDP-43, zebrafish, neurofilaments (NFs)

## Abstract

Neurofilaments (NFs), a major cytoskeletal component of motor neurons, play a key role in the differentiation, establishment and maintenance of their morphology and mechanical strength. The de novo assembly of these neuronal intermediate filaments requires the presence of the neurofilament light subunit (NEFL), whose expression is reduced in motor neurons in amyotrophic lateral sclerosis (ALS). This study used zebrafish as a model to characterize the NEFL homologue *neflb*, which encodes two different isoforms via a splicing of the primary transcript (*neflbE4* and *neflbE3*). In vivo imaging showed that *neflb* is crucial for proper neuronal development, and that disrupting the balance between its two isoforms specifically affects the NF assembly and motor axon growth, with resultant motor deficits. This equilibrium is also disrupted upon the partial depletion of TDP-43 (TAR DNA-binding protein 43), an RNA-binding protein encoded by the gene *TARDBP* that is mislocalized into cytoplasmic inclusions in ALS. The study supports the interaction of the NEFL expression and splicing with TDP-43 in a common pathway, both biologically and pathogenetically.

## 1. Introduction

A major cytoskeletal structure of an axon is composed by fibrillary networks of neurofilaments (NFs), which are expressed exclusively in mature neurons in both the central and peripheral nervous system [1,2,3]. NFs are formed by the assembly of three main proteins classified by their respective size: the neurofilament light (NEFL), middle and high proteins (NEFM and NEFH, respectively) [3]. All NF proteins possess a conserved central alpha-helical rod domain, necessary for the dimer formation during the first step of the assembly, and unique head and tail domains that protrude from the filament core [3]. The assembly properties of these factors depend on their stoichiometry [4,5] and post-translational modifications [6,7]. In fact, NEFM and NEFH cannot form long filaments by themselves, but each can co-assemble to form filaments in combination with NEFL both in vitro [8] and in cultured cells [9,10]. NFs are especially abundant in motor neurons (MNs), where they play a key role in the organization of the neuronal cytoarchitecture and are involved in perikarya differentiation, protrusion (dendrites and axons) maturation and synaptic functions [11].

Any defect in the NF assembly leads to dysfunction [12]. In particular, abnormal NF networks have been described in Charcot-Marie-Tooth disease (CMT) [13,14] and amyotrophic lateral sclerosis (ALS) [15].

In CMT, 30 mutations have been described in residues throughout NEFL [16] and cause variable clinical phenotypes [17] from the complete absence of NEFL [18] to the production of aggregate-prone mutants [19,20]. In particular, some of these mutants display altered phosphorylation patterns that suppress the filament assembly process, underlying the importance of this post-translational modification for the NF assembly [20]. In ALS, the abnormal deposition of hyper-phosphorylated forms of NFs has been detected in MNs [21,22,23]. The importance of NEFL in ALS is supported by the following evidence. First, high levels of the NEFL protein are detected in the cerebrospinal fluid (CSF) and blood of all ALS cases [24] as early as 12 months before the onset of the disease [25]. Consequently, NEFL is considered the most relevant biomarker in ALS and its level is used in ALS prognosis as it strictly correlates with the disease severity [26,27,28]. Second, the perturbation of the NEFL mRNA steady state in ALS spinal motor neurons could be a key pathogenic mechanism [29]. Indeed, disrupting the stoichiometry of the NF subunits leads to an NF aggregation reminiscent of the ALS pathology [30,31,32,33]. The evidence of a pathological dysregulation of the NEFL expression in ALS is strengthened by the existence of direct interactions between the NEFL mRNA and several ALS-causing proteins [34,35,36,37,38], including TDP-43 (TAR DNA-binding protein 43, encoded by the gene *TARDBP*), which has been shown to directly bind the human Nefl mRNA at its 3′UTR [39], and to induce ALS clinical traits through the mis-splicing of mRNA [40,41,42,43,44]. Importantly, the question of whether deficits in the NEFL RNA regulation can induce motor neuron degeneration has not been tested in vivo. In order to address this hypothesis, we used zebrafish (*Danio rerio*), a vertebrate model used to easily study normal and pathological events in the nervous system [45,46,47]. A number of genetic models in zebrafish have been established for a range of neurodegenerative disorders, including ALS [48,49,50,51,52,53,54].

**Figure 1:**
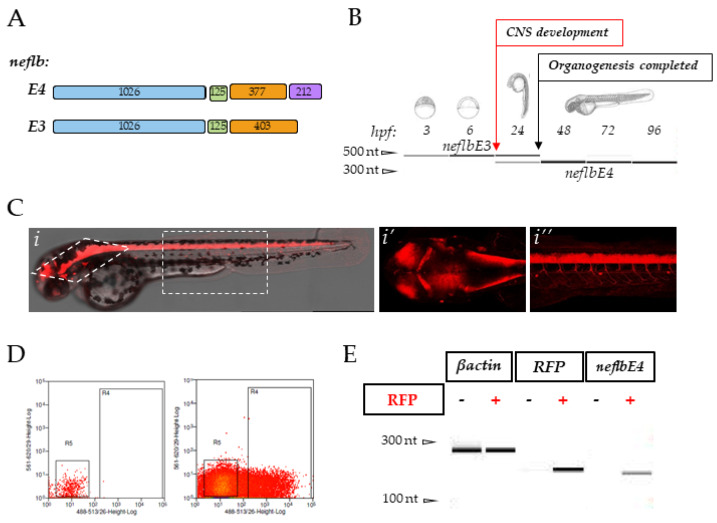
*neflbE3* and *neflbE4* expression in zebrafish. (**A**), schematic of the zebrafish exon structures of the predicted *neflb* splice variants. Length (bp) is indicated on each exon. (**B**), *neflb* is expressed at all embryonic and larval stages in zebrafish, with a splicing shift from *neflb3E* (upper PCR band) to *neflb4E* (lower PCR band) occurring during CNS development—revealed by a change in the amplicon size. (**C**) (*i–i″*), sagittal and dorsal vision of Tg(HuC:Gal4/UAS:RFP) zebrafish embryos at 48hpf. This transgenic line expresses the red fluorescent protein (RFP) in post-mitotic neurons. (**D**), post-mitotic neurons (RFP+) and non-neuronal cells (RFP-) were isolated by FACS, and the expression of β-actin, RFP and *neflb4E* was tested by PCR in both pools (**E**).

As of today, the zebrafish genome harbors a single predicted NEFL homolog, *neflb*, based on its sequence similarity with the human gene (Ensembl ID: ENSDARG00000012426). It displays about 60% of the nucleotide sequence identity with its mammalian homolog; the exon/intron structure has been mostly conserved through evolution, and two splice variants have been predicted for this gene (one with three exons—*neflbE3*—and one with four exons—*neflbE4*) in zebrafish (Figure 1A), as well as in humans (Ensembl ID: ENSG00000277586). *Neflb* has been previously shown to be important for CNS biology in zebrafish embryos [55].

In this study, we examined the consequences of altering the expression of this NEFL zebrafish orthologue and established a direct link between the *neflb* mRNA splicing modulations with an ALS-like phenotype (atrophy of motor axons and paralysis). We also explored the *neflb* expression in a TDP-43 knockdown model and the influence of the ectopic expression of the two *neflb* isoforms.

## 2. Material and Methods

### 2.1. Zebrafish Lines and Microinjections

Wild-type and transgenic zebrafish embryos were raised at 28 °C in an embryo medium: 0.6 g/L aquarium salt (Instant Ocean Spectrum Brands 3001 Commerce St. Blacksburg, VA 24060-6671) in reverse osmosis water containing 0.01 mg/L methylene blue. AB wild-type fish, and the transgenic lines Tg(Mnx1:eGFP) [56], Tg(elavl3:Gal4)zf349—referred to as Tg(HuC:Gal4)—[57], Tg(Mnx1:Gal4) [58] and Tg(5xUAS:RFP)^nkuasrfp1a^—referred to as Tg(UAS:RFP)—[59] have been used in this study. Zebrafish husbandry was performed according to approved guidelines. All procedures for zebrafish experimentation were approved by the Institutional Ethics Committee at the Research Center of the ICM and by French and European legislation. Animal facility of the Institut du Cerveau et de la Moelle épinière (ICM) received its accreditation from French authorities (Agreement #A75-13-19).

*neflb-eGFP constructs/cloning:* pUCminus containing N-terminally eGFP-tagged zebrafish cDNAs of both *neflb* splice variants (*neflbE3* and *neflbE4*) were purchased from Cliniscience. eGFP-*neflbE3* and eGFP-*neflbE4* were removed by restriction enzymes, and subcloned by ligation into pCS2 for a ubiquitous expression in SW13 cells and p5e-10xUAS [60] for the in vivo expression in zebrafish motoneurons using the Tg(Mnx1:Gal4) trigger line. 

*Gene knock down using antisense oligomorpholino oligonucleotides (Mo):* Antisense Mo were designed to complementarily bind to ATG or splice junctions that would block the transcription of the zebrafish targeted genes and synthesized from GeneTools (LLC 1001 Summerton Way Philomath, OR 97370 USA). The sequence of the previously described *TDP-43* Mo [48] is 5′-GTACATCTCGGCCATCTTTCCTCAG-3′. A splice-blocking antisense Mo against the *neflb* intron3-exon4 donor splice junction (*neflb*SV Mo) was synthesized (5′-CTCCCCTGTGAAAGAGTCAACAGAG-3′), and a standard control Mo—not binding anywhere in the zebrafish genome—was used to assess the specificity of the observed phenotypes (5′-CCTCTTACCTCAGTTACAATTTATA-3′).

An amount of 1 nL of DNA constructs (final concentration 75 ng/uL) and morpholino (0.2–0.6 mM) solutions were microinjected in one-cell stage embryos, using glass microcapillaries (Sutter Instrument) and a Picospritzer III (General Valve Corporation, Fairfield, NJ, USA) pressure ejector.

### 2.2. In Vivo Live Imaging and Timelapse

For the live confocal fluorescence imaging, embryos were mounted in a 1% low-melting point agarose (16520050; Invitrogen, Carlsbad, CA, USA) dissolved in embryo water and supplemented with 0.16 mg/mL tricaine (A-5040; Sigma, Saint-Louis, MO, USA). After solidification, the embryo medium with the 0.16 mg/mL tricaine solution was added in order to keep the embryos hydrated and anesthetized during the experiments. Thereafter, images were captured at the selected times on an inverted Leica SP8 set-up allowing a multiple point acquisition, and the same as for the image mutants and their siblings in parallel. Time-lapse acquisitions were captured with a spinning disk system (Andor Technology, Belfast, UK; Leica Microsystems, Germany), a DMI8 inverted stand (Leica Microsystems, Germany), a CSU-X head (Yokogawa, Japan) and a QE-180 camera (Hamamatsu, Japan), with a 20x objective (NA0.5). Image stacks were processed with the LAS software to generate maximum intensity projections or were exported into the ImageJ or Imaris software (Bitplane) for a 3D analysis of the motor axons projections.

### 2.3. Imaris Filament Tracer

For each embryo, a region of interest containing 4 somitic nerve fascicles (somites 13 to 16) was manually defined in 3D, then neurofilaments were analyzed using the filament tracer plugin of the Imaris software (Bitplane). The filament length, filament total volume, filament full branch depth and filament full branch level values were exported to Excel (Microsoft) and Prism (GraphPad).

### 2.4. Acridine Orange Staining

Apoptotic cells were revealed in 2-days-old live embryos by adding the acridine orange vital dye (N-4638; Sigma) to the embryo water at a final concentration of 5 mg/mL. Embryos were then incubated in the dark at 28 °C for 1.5–2 h, rinsed, anesthetized and observed under a fluorescent stereomicroscope Olympus (Shinjuku, Tokyo 163-0914, Japan).

### 2.5. Touch-Evoked Escape Response (TEER)

At 48 hpf, zebrafish embryos were tested for motor behavior under a stereomicroscope (Zeiss, Germany) using the touch-evoked escape response (TEER) experiment. Morphologically normal zebrafish from each experimental condition were touched lightly at the level of the tail with a tip, and their responses to the stimulus were recorded with a Grasshopper 2 Camera (Point Grey Research) at 30 Hz. The videos were then analyzed using the Manual Tracking plugin of the ImageJ software, and the swim duration, swim distance and maximum swim velocity of each embryo were calculated as previously described [49].

### 2.6. FACS (Fluorescence-Activated Cell Sorting)

Embryo dissociation was performed as previously described [61]. Sorted cells were collected in phosphate saline buffer (PBS) and RNA was extracted using Trizol reagent (T9424, Sigma, Saint-Louis, MO, USA). The extraction, RT and PCR were performed on three biological replicates of 20,000 cells each.

### 2.7. Cell Culture

The human cells SH-SY5Y were grown in D-MEM (Dulbecco’s modified Eagle medium; Gibco^®^, Life Technologies, Paisley, UK) supplemented with 10% fetal bovine serum and 1% of penicillin/streptomycin solution (100 U/mL). Cells were seeded at a density of 8 × 10^5^ cells on 35 mm tissue culture dishes and were transfected the following day with TDP-43 siRNA using Lipofectamine™ 2000 (Invitrogen™, Life Technologies, Paisley, UK) according to the manufacturer’s instructions. After 48 h of transfection, cells were washed with PBS and resuspended in 130 μL ice-cold extraction buffer: 50 mM Tris-HCl, pH 7.4/500 mM NaCl/5 mM EDTA/1% (*w/v*) Nonidet P-40/0.5% (*w/v*) Triton X-100 supplemented with a cocktail of protease inhibitors. Cell lysates were then sonicated and centrifuged at 14,000× *g* at 4 ºC for 20 min. The supernatants were collected and frozen at −80 °C until the biochemical analysis.

SW13vim-cells, which lack endogenous intermediate filaments, were cultured in Dulbecco’s modified essential medium with 5% fetal bovine serum (FBS). Cells were transfected with Lipofectamine 2000 in an Optimem medium (Invitrogen, Carlsbad, CA, USA) according to the manufacturer’s instructions using plasmids encoding EGFP-*neflbE4*, EGFP-*neflbE3*, mouse NEFM (identified by the clone NN18, Sigma–Aldrich, 1:1000) and NEFH (identified by the clone N52, Sigma–Aldrich, 1:1000), as previously described [62].

### 2.8. Reverse Transcription and PCR

The total RNA from zebrafish embryos, FACS-sorted cells or cell cultures was extracted using the TRIreagent (T9424, Sigma). RNA was quantified using the Nanodrop 8000 (Thermo Scientific, Waltham, MA, USA) and its quality was checked using the 2100 Bioanalyzer (Agilent Technologies, Santa Clara, CA, USA). cDNA was synthesized from 1 ug of RNA using Transcriptor Universal cDNA Master Mix (Roche). PCR experiments were performed to assess the expression of genes using primer pairs amplifying the sequences of interest (Supplementary Table S1).

### 2.9. Western Blot

Embryos at the stage of interest were deeply anesthetized, and collected into 1.5mL microcentrifuge tubes. The water and anesthetic were completely removed and replaced by 200uL of RIPA solution supplemented with protease inhibitors. Tissue dissociation was performed by ultrasound, and solubilized proteins were quantified with the Pierce BCA protein assay (Thermofisher).

Proteins were denatured for 7 min at 98 °C, separated on 4–12% bis-Tris Gel (NuPAGE), transferred on nitrocellulose membranes (Whatman Protran), blocked with 5% BSA in Tris-buffered saline (TBS) buffer, incubated with Tubulin (T5168, Sigma) or TDP-43 (3449S, Cell Signaling) antibodies diluted in TBS plus 5% BSA, washed three times, and incubated with anti-rabbit or anti-mouse dye-conjugated antibodies (1:3,000, Cell Signaling) for 1 h in TBS, followed by washing, and the signal was detected using the ODYSSEY^®^ CLx. The intensity of the bands was measured by the open source image processing program ImageJ.

### 2.10. Statistical Analysis

Data were plotted and analyzed using the Excel software (Microsoft, Redmond, WA, USA). The Prism (Graphpad) software was used for the statistical analysis. Details of each test are described in the related figure legends. 

## 3. Results

### 3.1. The Zebrafish Homolog neflb Encodes Two Splice Variants, neflbE3 and neflbE4, Differently Expressed during the Development

In order to study the *neflb* expression through gastrulation and organogenesis, we extracted the RNA from the zebrafish embryos at 3, 6, 24, 48, 72 and 96 h post fertilization (hpf), and designed primers so to reveal both splice variants and differentiate them by their size. As shown in Figure 1B, *neflb* is expressed in zebrafish at all the tested stages, and both expected splice variants were detected. *neflbE3* (upper PCR band) was detected only from 3 up to 24 hpf, while the other variant, *neflbE4* (lower PCR band), was expressed from 24 hpf. This temporal shift in expression correlates with neurogenesis and with the stage at which the *neflb* mRNA, detected by the in situ hybridization, stopped being expressed ubiquitously, and started being expressed specifically in developing neurons [55]. To determine whether the *neflbE4* isoform was indeed specifically expressed in neuronal cells, we used Tg(HuC:Gal4/UAS:RFP) embryos. In this double transgenic line, all post-mitotic neurons (HuC positive) express the red fluorescent protein (RFP) (Figure 1C,(*i–i*”)); after the FACS-sorting of the RFP+ neurons from the RFP non-neuronal cells (Figure 1D), RNA was isolated and PCR was performed to detect mRNA for the genes of interest with β-actin as a control. As shown in Figure 1E, both cell pools express similar levels of β-actin mRNA, whereas the RFP mRNA was only expressed in the neuronal pool. *neflbE4* mRNA was detected only in the post-mitotic neurons.

### 3.2. neflbE3/E4 Misbalance Results in a Strong and Specific Motor Phenotype

**Figure 2:**
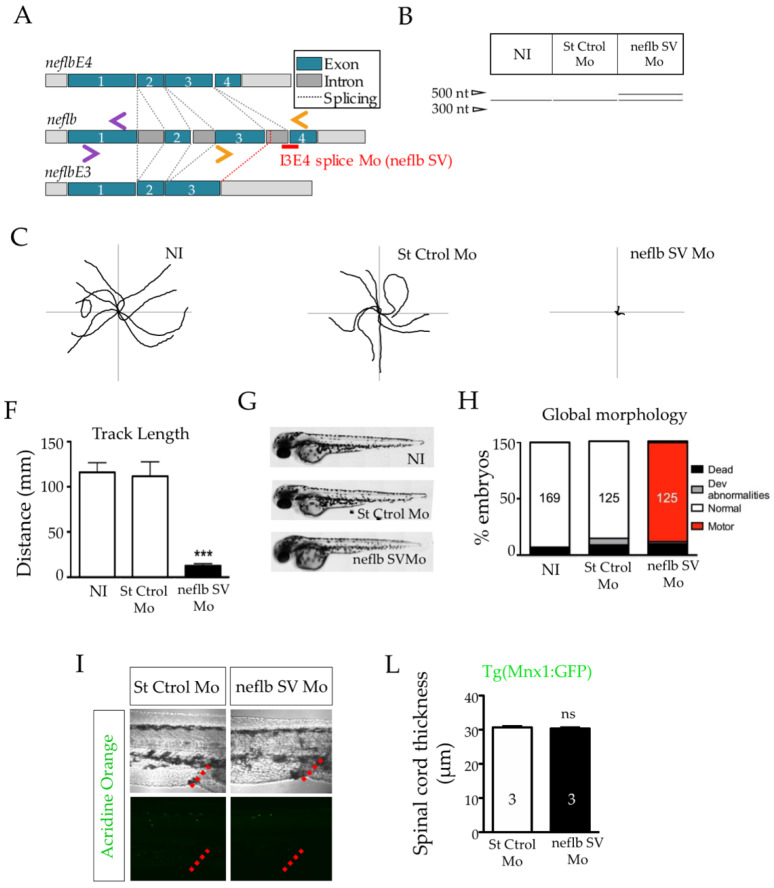
*neflbE3/E4* misbalance results in a strong and specific motor phenotype. (**A**), position of the splicing variant (SV) antisense oligomorpholino (neflb SV Mo, in red) targeting the decisive I3E4 splice junction in order to inhibit the developmental shift from *neflb3E* to *neflb4E*, and of the primer pairs used in this study: ZF Neflb (purple pair) and ZF Neflb SV (orange pair). (**B**), St Ctrol Mo and neflb SV Mo were injected at the dose of 0.2 mM. The I3E4 morpholino was efficient and caused the persistence of the *neflb3E* (upper band) expression at 48 hpf, when only *neflb4E* (lower band) was expressed in controls at this stage. (**C**), trajectories of 9 representative zebrafish embryos per condition during the touch-evoked escape response at 48 hpf. Non-injected (NI) and control injected embryos (St Ctrol) swam to the edges of a Petri dish in reaction to a light touch; neflb SV morphants were unable to move away from the center of the dish. (**F**), quantification of the track length shows a reduction by 90% in the swimming distance in neflb SV Mo-injected fish compared with the controls. (**G**), neflb SV Mo-injected embryos develop without any major developmental abnormality. (**H**), bar graph of the phenotype distribution after the TEER analysis at 48 hpf. Percentages of zebrafish with motor deficits (motor) are increased after the neflb SV Mo injection. Percentages of normally developed embryos in NI and St Ctrl Mo conditions are comparable. (**I**), Acridine orange staining (green), which is a metachromatic intercalator sensitive to DNA conformation, is used in this study to detect apoptosis. No higher amount of acridine orange-positive cells was detected in the spinal cord of the neflb SV morphants (right panel) compared with the controls (left panel). The urogenital opening (red dotted lines) has been used as a geographical reference for the measures. (**L**), spinal cord thickness measured at 48 hpf. *** *p* < 0.001; ns, non-significant.

In order to study the role of *neflb* splicing and the importance of *neflbE4* in CNS development, we designed an antisense morpholino oligonucleotide (Mo) targeting the splice acceptor junction between intron 3 and exon 4 of the *neflb* gene (*neflb* SV Mo) (Figure 2A). 

This Mo was predicted to prevent the excision of intron 3, driving the splicing balance towards the generation of the *neflbE3* isoform. As expected, at 48 hpf, only *neflbE4* (Figure 2B, lower band) was detected in the control uninjected embryos, as well as in embryos injected with a standard control Mo (St Ctrol Mo). In contrast, in embryos injected with *neflb* SV Mo, an upper band appeared representing the splicing variant *neflbE3*, whose expression is maintained beyond 24 hpf. 

We then characterized the motor phenotype of each condition at 48 hpf by a touch-evoked escape response (TEER). Following a light touch, both uninjected and St Ctrol Mo-injected embryos escaped and swam away from the center of the Petri dish, all the way to the edges of the plate, whereas embryos injected with *neflb* SV Mo did not (Figure 2C), with the swimming distance being reduced by 89% (Figure 2F).

Although no developmental abnormalities were observed among conditions (Figure 2G), the *neflb* SV Mo injection resulted in a very strong and specific motor phenotype (88% of embryos) characterized by the inability to swim (Figure 2H). As no differences were observed between the uninjected and St Ctrol embryos, only the latter were used further in this study.

To determine whether the motor deficit observed was associated with cell death, acridine orange vital staining was used to detect apoptotic neurons. No major signal was detected in the *neflb* SV Mo embryos compared with controls (Figure 2I). There were also no significant differences in the spinal cord thickness among the groups (Figure 2L). Thus, we concluded that the abnormal expression of the *neflbE3* isoform does not affect neuron development.

### 3.3. The Motor Phenotype Associated with the Alteration of neflbE3 Expression Correlates with Atrophy of Motor Axons

**Figure 3:**
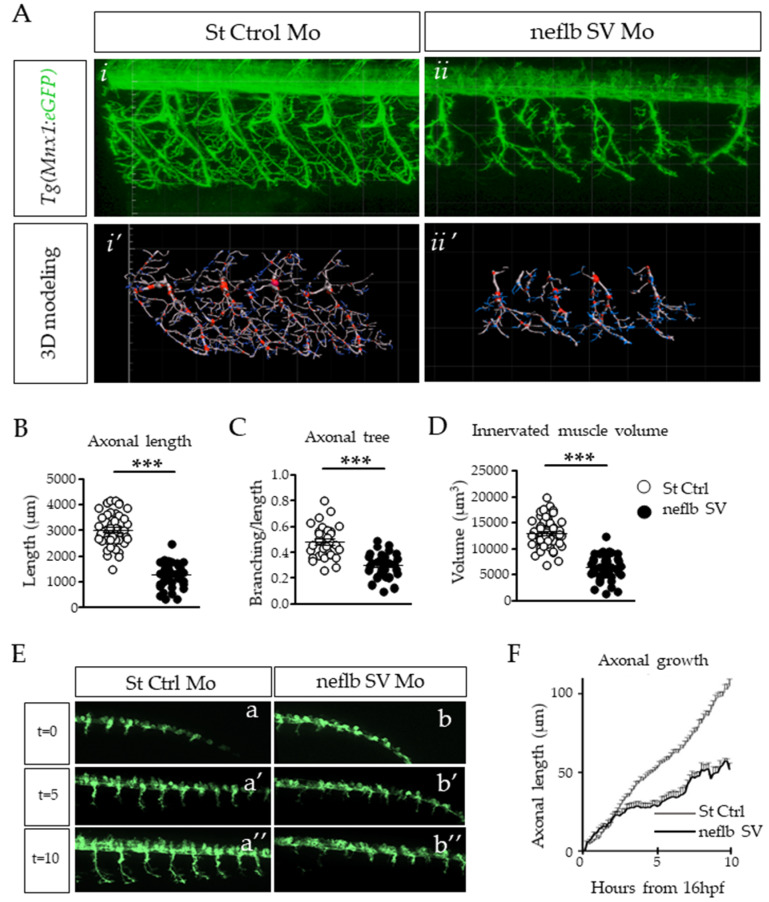
Axonal atrophy in motor neurons of neflb SV embryos. (**A**), in vivo observation of somitic nerve fascicles in 48 hpf Hb9:eGFP zebrafish embryos injected with a control Mo (*i*) or with neflb SV Mo (*ii*). Hb9:eGFP line express the GFP (green) in motor neurons under the specific promoter HB9. After the neflb SV Mo injection, the axonal projections appeared shorter and less regularly distributed and branched than controls. (*i’*,*ii’*), 3D modeling of motor neuron morphology using the Imaris software. This reconstruction permitted the quantitative analysis of the nerve fascicles features: (**B**), the axonal length; (**C**), the axonal branching; (**D**), the volume of the innervated muscle. All measurements were reduced in the neflb SV morphants (black circles) with respect to the controls (white circles). (**E**), in vivo time-lapse imaging of the *Tg(Mnx1:eGFP)* zebrafish embryos injected with a control morpholino (a–a″) or with the neflb SV morpholino (b–b″) was performed during 10 h, starting from 16 hpf. (**F**), quantification of motor neurons axonal length during time. hpf, hour post fertilization. *** *p* < 0.001.

Previous work has shown a direct link between motor behavior deficits and the disorganization of motor neuron morphology [49,63,64,65]. Thus, we examined the somitic axonal projections of the motor neurons. Axonal projections from motor neurons exit the spinal cord grouped as one nerve fascicle per somite, and then grow along the somitic muscle all the way to its most ventral part and then back up around it, while branching and connecting with muscle fibers. Mirror innervation of the dorsal part of the somitic muscle is achieved through the same process. As shown in Figure 3A(*i*), all somitic muscles appeared properly innervated in the control embryos.

However, the neflb SV morphants displayed shortened and disorganized motor neuron nerve fascicles with major branching abnormalities (Figure 3A(*ii*)). The filament tracer plugin of the Imaris software was used to model the nerve fascicles in 3D and analyze the morphological defects (Figure 3A(*i’*,*ii’*)). In the neflb SV morphants, the somitic nerve fascicle main length was decreased by 46% as compared with controls (Figure 3C). Furthermore, axons were less branched (Figure 3D) and innervated a reduced area on the muscle (Figure 3E), confirming the strong and specific phenotype of the neflb SV morphants.

To refine our understanding of this phenotype, we performed in vivo time-lapse imaging over the first ten hours (starting from 16 hpf) of the muscle innervation by the motor axons, in controls and in the nefl SV morphants in parallel. As shown in Supplementary Movie 2 and in Figure 3E, the motor axonal projections in the *nefl* SV morphants appear to initiate at the same developmental stage as in the controls (Figure 3E(a–b’) and Supplementary Movie 1), and they sprout in the right direction, ventrally towards the somitic muscles. However, as shown in Supplementary Movie 2, the motor axons constantly grew back and forth, and never reached a normal length (Figure 3E(a″–b″),F).

### 3.4. neflbE4 Participates in Motor Neuron Axonal Growth, Whereas neflbE3 Fails to Polymerize Normally and Forms Aggregates

In order to assess the assembly properties of *neflbE4* and *neflbE3* in vivo, we generated both UAS: eGFP-*neflbE4* and UAS:eGFP-*neflbE3* constructs. These constructs were separately injected into Tg(Mnx1:Gal4/UAS:RFP) zebrafish embryos, in order to target the expression of the two isoforms in the motor neurons. 

**Figure 4:**
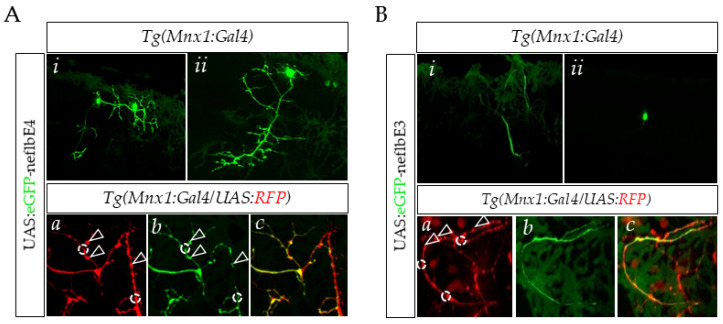
neflbE4 and neflbE3 distribution within motor neurons. (**A**) (*i*,*ii*), in vivo observation of single motor neurons expressing eGFP-neflbE4. Motor neurons expressing eGFP-neflb4E extend long and ramified axons. *(a–c*), eGFP-neflbE4 is particularly abundant in the specific area representing the axonal branching (white dotted circles) and NMJ buttons (white arrows). (**B**) *(i*,*ii*), single motor neurons expressing eGFP-neflb3E. eGFP-neflbE3 mostly aggregated or assembled into a long, massive, unramified bundle. *(a–c*), eGFP-neflbE3 was reduced or absent in the axonal ramification sites and NMJ buttons.

At 48 hpf, the eGFP-*neflbE4*-positive motor neurons have the same morphology as in the eGFP+ controls (Supplementary Figure S1A). The eGFP-*neflbE4* protein is homogenously distributed throughout the cell bodies, axons and dendrites, (Figure 4A(*i*,*ii*)) and is abundant in all axonal ramifications (Figure 4A(*a–c*), dotted circles) and neuromuscular junction (NMJ) boutons (Figure 4A(*a–c*), arrows). In contrast, the expression of the eGFP-*neflbE3* isoform induced changes in the motor neuron morphology, with the loss of dendrite extensions. In addition, the eGFP-*neflbE3* isoform formed cytoplasmic bundles (Figure 4B(*i*)) and aggregates (Figure 4B (*ii*)) in almost all motor neurons expressing the construct (Supplementary Figure S1B) and impaired the formation of axonal ramifications and NMJ (Figure 4B(*a*–*c*)). 

### 3.5. neflb Splice Variants Have Different Assembly Properties in SW13^vim-^ Cells

**Figure 5:**
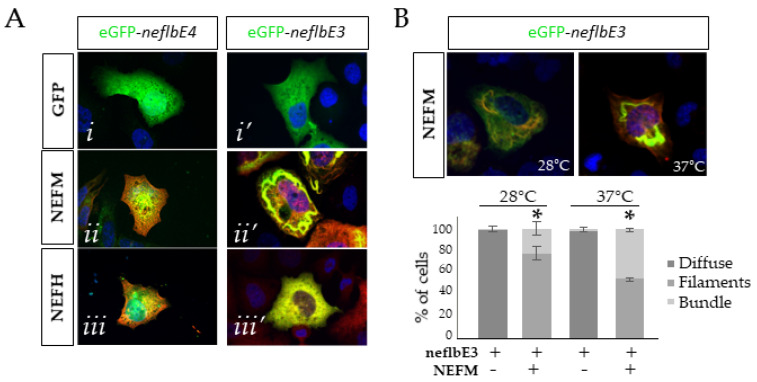
The *neflb* splicing variants assembly properties in SW13vim cells. (**A**), eGFP-neflbE4 (*i*) and eGFP-neflbE3 (*i’*) were expressed in SW13vim cells alone or together with the mouse NEFM (*ii*,*ii’*, red) or NEFH (*iii*,*iii’*, red). The assembly pattern was characterized at 48 h post transfection at 37 °C. (**B**), eGFP-neflbE3 and NEFM were co-transfected and the bundle formation was observed at 37 °C and 28 °C. *Bottom panel*, bar graph showing the percentage of cells carrying filaments, bundles or diffuse labeling. At 28 °C, the proportion of cells carrying bundles made of neflb3E and NEFM was less than the proportion of cells carrying bundles at 37 °C. * *p* < 0.05, (*n* = 3 cultures, 60 to 100 cells per coverslip) one-way ANOVA Tukey’s HSD post-hoc analysis.

To evaluate the assembly properties, the constructs were expressed in SW13^vim-^ cells, which lack endogenous intermediate filament proteins. In mammalian cells, NEFL dimerizes with NEFM and NEFH and is a core protein necessary for the assembly of NF proteins into filamentous structures [66]. In order to determine the assembly competency of the *neflb* variants, eGFP-*neflbE4* or eGFP-*neflbE3* were expressed alone or in combination with the mouse NEFM or NEFH (Figure 5A, Supplementary Figure S2).

Neither *neflbE4* nor *neflbE3* assembled into filamentous structures on their own (Figure 5A(*i*,*i*’)). Although eGFP-*neflbE4* and eGFP-*neflbE3* partially co-localized with NEFM, suggesting an interaction, only eGFP-*neflbE3* could form higher order structures (filaments and bundles) with the NEFM (Figure 5A(*ii*’)). Finally, neither of them formed filaments in the presence of NEFH (Figure 5A(*iii*,*iii*’), Supplementary Figure S2).

As zebrafish are poikilothermic organisms living at 28 °C, *neflb* could have different assembly properties according to the temperature, as previously shown for the lamprey Nefl [67]. Therefore, the assembly of *neflbE3* with NEFM was verified not only at 37 °C but also at 28 °C (Figure 5B). At 28 °C, *neflbE3* was still able to form bundles of filament with NEFM, although less efficiently than at 37 °C. Thus, the basic mechanisms of assembly and bundling of *neflbE3* are operant at both temperatures. 

### 3.6. TDP-43 Regulates neflb Splicing in Zebrafish

As TDP-43 mutations are known to induce ALS clinical traits through the mis-splicing of mRNA [40,41,42,43,44] and considering that it has been shown to directly bind to human Nefl mRNA at its 3′UTR [39], we aimed to determine if *neflb* splicing occurs in the case of a morpholino-driven TDP-43 depletion, which is a well-characterized zebrafish model of ALS [48]. The knockdown efficiency of the TDP-43 expression was measured by Western blot analysis (Supplementary Figure S3A) and zebrafish showed a significant reduction in the swimming parameters at 48 hpf of TDP-43 knockdown (KD) (Supplementary Figure S3B) as well as decreased somitic muscle innervation (Supplementary Figure S3C).

**Figure 6:**
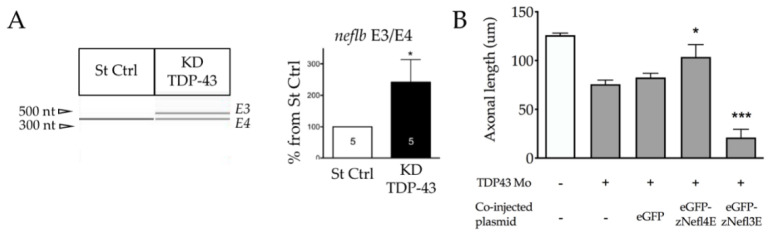
TDP-43 regulates *neflb* splicing in zebrafish. (**A**) left, PCR analysis on RNA extracted from St Ctrl embryos and fish KD for TDP-43, using specific primers for both isoforms *neflbE4* and *neflbE3*. Right, quantification of the *neflbE3* and *neflbE4* ratio. (**B**), axonal length measurement at 48 hpf after the overexpression of eGFP-*neflbE4* and eGFP*-neflbE3* constructs in KD TDP-43 embryos. *neflbE4* significantly rescued the axon length (middle histo bar), whereas *neflbE3* significantly aggravated the KD TDP-43 phenotype (right histo bar). * *p* < 0.05, *** *p* < 0.001, in comparison to TDP-43 Mo + eGFP.

Interestingly, in the absence of TDP-43, *neflb* intron 3 was retained (Figure 6A), suggesting an important role for TDP-43 in the regulation of the NF expression and maintenance of the NF protein stoichiometry, with a consequent impact on the motor neuron morphology and function.

To verify this hypothesis, we injected the previously described eGFP-*neflbE4* and eGFP-*neflbE3* constructs into the TDP-43 KD embryos expressing Tg(Mnx1:Gal4) and compared the motor neuron axonal length among the conditions (Figure 6B). Interestingly, the reduced axonal length of the motor neurons of the TDP-43 KD embryos (eGFP) can be rescued upon an eGFP-*neflbE4* injection, showing that *neflb* splicing is a key event in the pathogenic cascade leading to an abnormal motor neuron architecture. Importantly, the eGFP-*neflbE3* isoform expression exacerbated such a deficit, showing that the proper splicing of *neflb* is key for motor neurons.

## 4. Discussion

The post transcriptional regulation of *NEFL* can have a wide impact on the NF network. However, the relevance of *NEFL* mRNA metabolism in ALS pathogenesis is unclear. In this study, we addressed the function of zebrafish *neflb* processing in the regulation of motor neuron development and the pathogenic consequences of dysregulation. Only recently, the *NEFL* orthologue *neflb* has been characterized in zebrafish and shown to be expressed in neurons [55]. Here, we described the existence of two mRNA splice variants of this gene that we called *neflbE4* and *neflbE3*, which are differently expressed during zebrafish development. *neflbE3* is the first isoform to be produced ubiquitously before being completely replaced by *neflbE4*, which is a neuron-specific isoform. Interestingly, when the *neflbE3* isoform expression was forced to persist beyond its normal developmental window, the fish displayed a strongly compromised swimming motor behavior, which correlated with the aberrant motor axons’ growth and ramification. Importantly, these affected motor neurons present an abnormal assembly of the *neflbE3* protein consistent with the formation of abnormal bundles and aggregates when expressed together with NEFM in SW13^vim^, the human cell line lacking intermediate filaments commonly used to study a de novo NF assembly. 

The requirement for NEFL in the assembly of the other subunits, NEFM and NEFH, has been widely described [5,68,69]. Thus, Nefl KO mice exhibit motor neurons with no NFs and axons of a smaller caliber [70], as well as functional consequences [71]. Furthermore, *NEFL* mRNA is reduced in ALS motor neurons in autopsy specimens and is associated with an altered NF triplet protein stoichiometry and neurofilamentous aggregation [29]. 

The relevance of the disruption of the NF stoichiometry has also been implicated in conditions of an NEFL overexpression. The transgenic overexpression of Nefl in mice was sufficient to generate an NF accumulation in the motor neuronal perikarya and proximal axons, accompanied by axonal and dendritic degeneration [31]. 

On the contrary, the additional expression of NEFL was beneficial in a transgenic mouse overexpressing NEFH [72], reducing the NF accumulation and axonal transport defects in a dose-dependent manner. Taken together, these results reinforce the importance of the precise stoichiometric abundance of NEFL for a proper NF assembly. 

In our zebrafish model, the re-expression of the early isoform of *neflb* (*neflb3E*) at the later stages induced motor and morphological phenotypes reminiscent of what is observed in transgenic mice and ALS patients. The ensemble of our results confirm the importance of NFs’ stoichiometry for polymerization and the existence of splicing mechanisms in the regulation of the NF biology in zebrafish.

mRNA mis-splicing is a common pathological mechanism widely recognized in ALS. In particular, the effects of TDP-43 on RNA metabolism have been extensively studied due to the pivotal role that this protein plays in ALS physiopathology [40,41,42,43,44]. TDP-43 mutations are known to induce ALS clinical traits through mRNA mis-splicing in patients [73,74]. The depletion of TDP-43 in a cell culture can cause the abnormal splicing of cryptic exons and lead to toxicity [42]. Further, mice expressing TDP-43 mutated in its low-complexity domain suffered from neurodegeneration that correlated with the consistent skipping of the constitutive exons normally spliced by the wild-type TDP-43 (named skiptic exons) [44,75]. In 2007, the direct stability-mediated effect of TDP-43 on *NEFL* mRNA was described [39]. In this study, we explored the influence of TDP-43 on the *neflb* gene expression in zebrafish using an ALS model generated by *Tardbp* KD [48]. The TDP-43 loss of function induced a retention of intron 3 of the zebrafish *neflb* gene, with the consequent generation of the neflbE3 isoform. Importantly, the overexpression of *neflbE4* ameliorated the impairment of the motor neuron axonal length in TDP-43 KD mice, whereas the *neflbE3* overexpression aggravated this phenotype. These observations, together with the overlapping motor and morphological deficits support the interaction of TDP-43 and *nefl* in a common pathway, both biologically and pathogenetically.

In terms of the mammalian NEFL, two possible splice variants are predicted for this transcript (Ensembl, ENSG00000277586) [76,77]. This prediction is corroborated by the presence of multiple NEFL bands on the Western blots of neuronal tissues, but they remain poorly characterized [78]. 

Altogether, the possible existence of a physiological or pathological splicing regulation of the human NEFL and relevance to disease should be explored in light of the results of this study in zebrafish. 

## Supplementary Materials

The following are available online at https://www.mdpi.com/2073-4409/9/5/1238/s1.
