# Supplementary material for: Functional Characterization of Neurofilament Light Splicing and Misbalance in Zebrafish
Source: Cells. 2020 May 16;9(5):1238. doi: 10.3390/cells9051238 (PMC7291018; doi:10.3390/cells9051238)
Supplement: Supplementary file 1 [file cells-09-01238-s001.zip › Neflb Supp Figures/Supplementary Figure 1.docx]

**
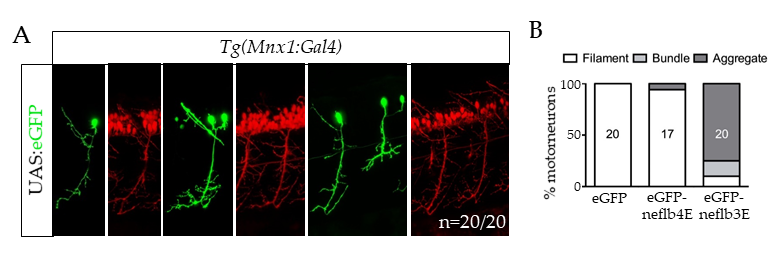
**

**Supplementary Figure 1. A*,*** *in vivo* observation of single motor neurons expressing eGFP from a UAS:eGFP plasmid injected at the 1-cell stage. Motor neurons expressing eGFP extend long and ramified axons. eGFP was detected in the whole cell, body, axon and ramifications, and did not induce silencing of the RFP reporter in any of the analyzed cells (n=20). **B**, quantification of percentages of filaments, bundles and aggregates structures developed in single motor neurons expressing eGFP-zNefl4E or eGFP-zNefl3E.
