# Supplementary material for: Functional Characterization of Neurofilament Light Splicing and Misbalance in Zebrafish
Source: Cells. 2020 May 16;9(5):1238. doi: 10.3390/cells9051238 (PMC7291018; doi:10.3390/cells9051238)
Supplement: Supplementary file 1 [file cells-09-01238-s001.zip › Neflb Supp Figures/Supplementary Figure 2.docx]

***
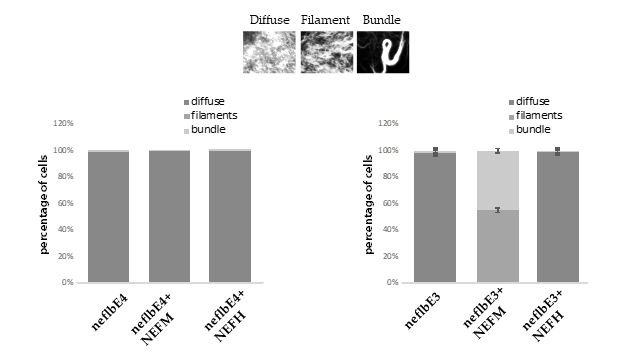
* Supplementary Figure 2.** Graphical representation of the percentage of cells carrying bundles, filaments and diffuse NFs structures in SW13^vim-^ cells transfected with eGFP-neflbE4 (left) and eGFP-neflbE3 (right) alone or co-expressed with NEFM or mouse NEFH. The upper panel shows the typical shape of filaments, bundles or diffuse labeling that have been detect in transfected cells and quantified.
