# Supplementary material for: Functional Characterization of Neurofilament Light Splicing and Misbalance in Zebrafish
Source: Cells. 2020 May 16;9(5):1238. doi: 10.3390/cells9051238 (PMC7291018; doi:10.3390/cells9051238)
Supplement: Supplementary file 1 [file cells-09-01238-s001.zip › Neflb Supp Figures/Supplementary Figure 3.docx]

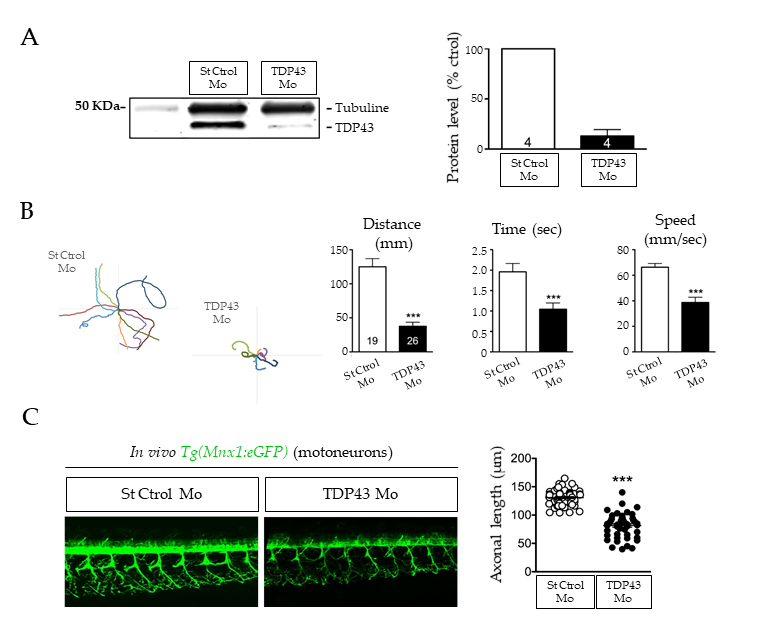


**Supplementary Figure 3:** **zTDP43 knock-down results in motor phenotype and axonal atrophy in zebrafish embryos.** **A**, TDP43 morpholino injection in zebrafish resulted in an almost 90% reduction of the TDP43 protein, as shown by WB. This KD lead to a strong and specific motor phenotype, as TDP43 morphants were unable to swim to the edges of a petri dish during TEER (**B**, left panel). Their displacement was shorter in distance, time and speed (B, right panel). **C**, motor axons of TDP43 morphants, revealed by GFP expression under the Mnx1 promoter *in vivo*, were shorter than control fish injected with ST Ctrol Mo. ***P<0.001.
