# Supplementary material for: Functional Characterization of Neurofilament Light Splicing and Misbalance in Zebrafish
Source: Cells. 2020 May 16;9(5):1238. doi: 10.3390/cells9051238 (PMC7291018; doi:10.3390/cells9051238)
Supplement: Supplementary file 1 [file cells-09-01238-s001.zip › Neflb Supp Figures/Supplementary Table 1.docx]

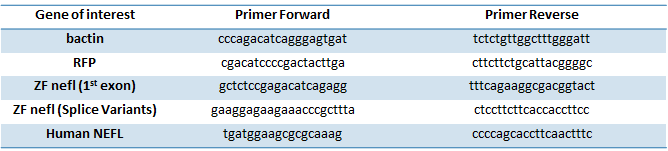


**Supplementary Table 1 : Primer sequences used for PCR**

For position of the zebrafish Nefl primers, see Fig 2A
