# Supplementary figures and images for: Functional Characterization of Neurofilament Light Splicing and Misbalance in Zebrafish
Source: Cells. 2020 May 16;9(5):1238. doi: 10.3390/cells9051238 (PMC7291018; doi:10.3390/cells9051238)

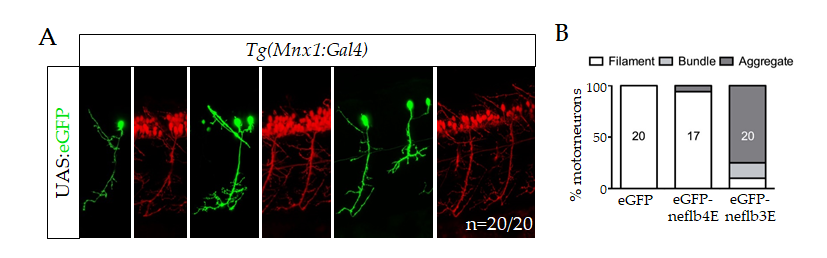

Supplement: Supplementary file 1 [file cells-09-01238-s001.zip › Neflb Supp Figures/Sup fig1.tif]

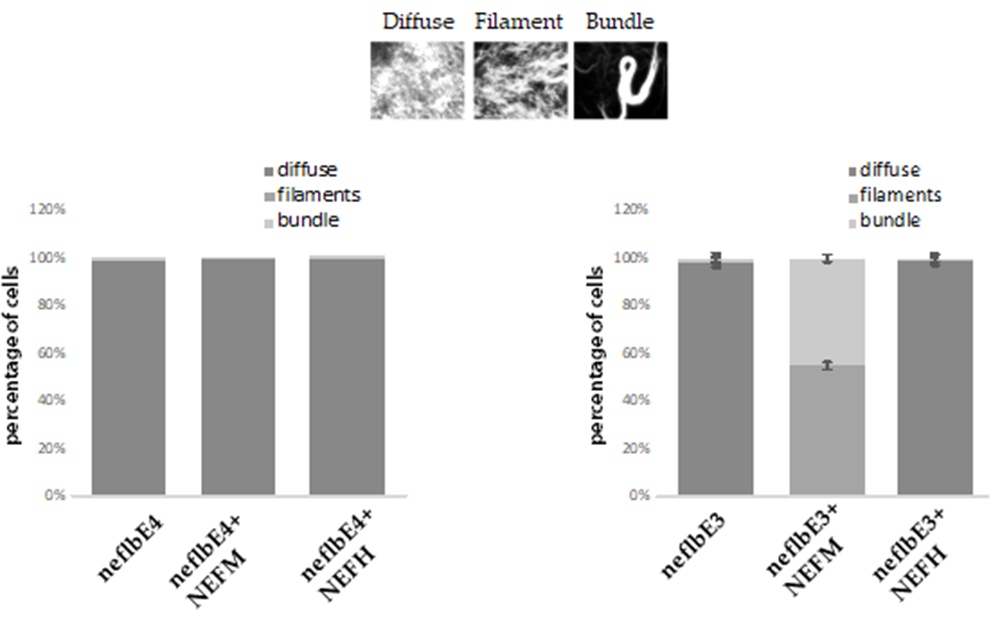

Supplement: Supplementary file 1 [file cells-09-01238-s001.zip › Neflb Supp Figures/Sup fig2.tif]

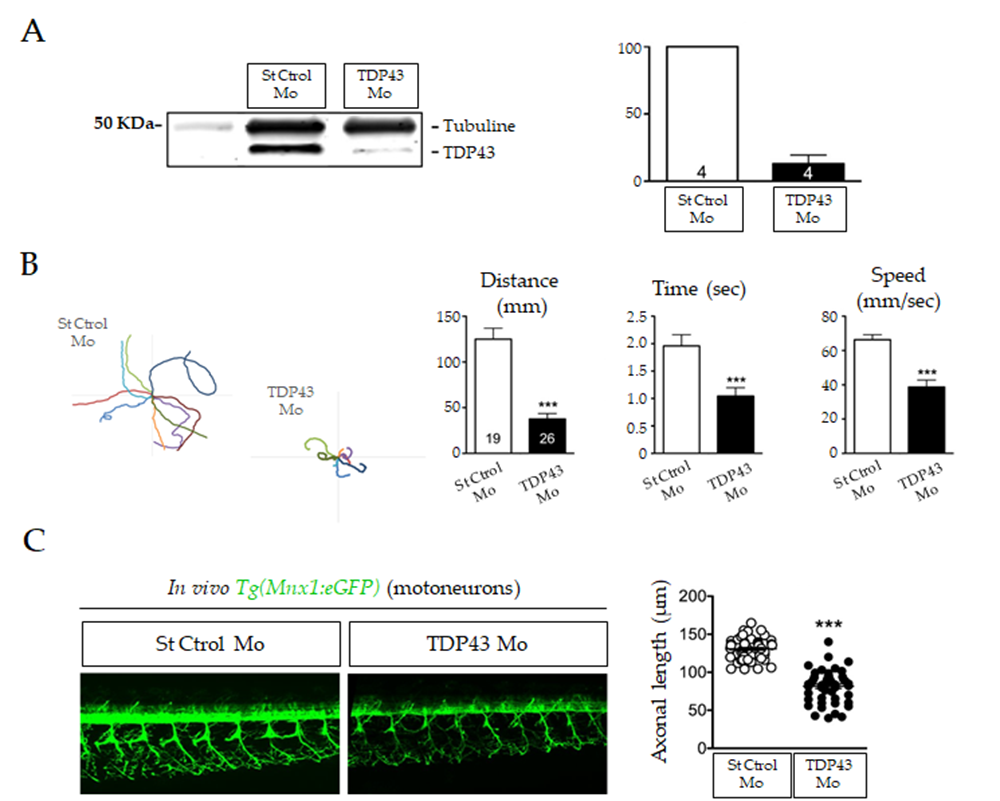

Supplement: Supplementary file 1 [file cells-09-01238-s001.zip › Neflb Supp Figures/Sup fig3.tif]

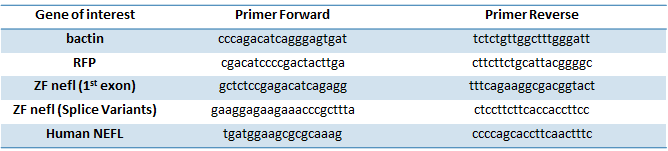

Supplement: Supplementary file 1 [file cells-09-01238-s001.zip › Neflb Supp Figures/Sup table.tif]
